# Sudden cardiac arrest in athletes and strategies to optimize preparedness

**DOI:** 10.1002/clc.24095

**Published:** 2023-07-26

**Authors:** Aneeq Malik, Justin Hanson, Janet Han, Brett Dolezal, Jason S. Bradfield, Noel G. Boyle, Jeffrey J. Hsu

**Affiliations:** ^1^ Department of Medicine Olive View‐UCLA Medical Center Los Angeles California USA; ^2^ Department of Medicine, Division of Cardiology David Geffen School of Medicine at UCLA Los Angeles California USA; ^3^ Department of Medicine, Division of Cardiology Veterans Affairs Greater Los Angeles Healthcare System and UCLA Los Angeles California USA; ^4^ Exercise Physiology Research Laboratory, Departments of Medicine and Physiology David Geffen School of Medicine at UCLA Los Angeles California USA; ^5^ UCLA Cardiac Arrhythmia Center David Geffen School of Medicine at UCLA Los Angeles California USA

**Keywords:** AED, athletes, CPR, emergency action planning, preparticipation screening, sudden cardiac arrest, sudden cardiac death

## Abstract

Sudden cardiac arrest (SCA) is the leading cause of death in young athletes. Despite efforts to improve preparedness for cardiac emergencies, the incidence of out‐of‐hospital cardiac arrests in athletes remains high, and bystander awareness and readiness for SCA support are inadequate. Initiatives such as designing an emergency action plan (EAP) and mandating training in cardiopulmonary resuscitation (CPR) and automated external defibrillator use (AED) for team members and personnel can contribute to improved survival rates in SCA cases. This review provides an overview of SCA in athletes, focusing on identifying populations at the highest risk and evaluating the effectiveness of different screening practices in detecting conditions that may lead to SCA. We summarize current practices and recommendations for improving the response to SCA events, and we highlight the need for ongoing efforts to optimize preparedness through the implementation of EAPs and the training of individuals in CPR and AED use. Additionally, we propose a call to action to increase awareness and training in EAP development, CPR, and AED use for team members and personnel. To improve outcomes of SCA cases in athletes, it is crucial to enhance bystander awareness and preparedness for cardiac emergencies. Implementing EAPs and providing training in CPR and AED use for team members and personnel are essential steps toward improving survival rates in SCA cases.

## Graphical abstract

Emergency action plan (EAP) checklist. Checklist for creating a personalized EAP in the event of a sudden cardiac arrest (SCA). This includes requirements for on‐site personnel, off‐site personnel, emergency equipment, and rehearsal of the EAP. These are general recommendations following an SCA; site‐specific changes can be made to ensure proper emergency response.

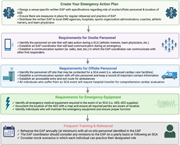

**Abbreviations**
AAU: amateur athletic union
AED: automated external defibrillator
AHA: American Heart Association
AN SUD: autopsy negative sudden unexplained death
ARVC: arrhythmogenic right ventricular cardiomyopathy
AY: athlete years
BrS: Brugada syndrome
CARES: Cardiac Arrest Registry to enhance survival
CPR: cardiopulmonary resuscitation
CPVT: catecholaminergic polymorphic ventricular tachycardia
EAP: emergency action plan
ESC: European Society of Cardiology
FIFA: Fédération Internationale de Football Association
FIFA‐SDR: FIFA Sudden Death Registry
HCM: hypertrophic cardiomyopathy
IOC: International Olympic Committee
NCAA: National Collegiate Athletic Association
OHCA: out‐of‐hospital cardiac arrest
PEA: pulseless electrical activity
SCA: sudden cardiac arrest
SCD: sudden cardiac death
VF: ventricular fibrillation
VT: ventricular tachycardia

## 1 INTRODUCTION

Sudden cardiac arrest (SCA) is the leading medical cause of death in athletes and can occur regardless of age and physical conditioning.^1^ For those over the age of 35, atherosclerotic coronary artery disease accounts for the majority of deaths during physical activity.^2^ Younger athletes at risk for SCA during exercise are those with underlying cardiomyopathies or inherited arrhythmia syndromes.^2^ Regardless of the underlying condition, these potentially fatal cardiac events occur most often during exercise. Exercise has frequently been shown to reduce the long‐term risk of cardiovascular disease,^3^, ^4^, ^5^ while at the same time, strenuous exercise acutely increases the risk of SCA.^6^, ^7^, ^8^ This “Exercise Paradox” illustrates how exercise can provide long‐term protection against cardiovascular events while also acutely predisposing a vulnerable individual to a relatively increased risk of SCA.^9^ Habitual exercise is expected to enhance the electrical stability of the myocardium, protecting against ventricular arrhythmias and SCA, while episodes of vigorous exercise may activate the sympathetic nervous system and trigger ventricular arrhythmias in the presence of susceptible myocardial substrate.^10^

This paradox may explain why athletes considered to be at the peak of physical fitness and one of the healthiest segments of our society can still experience SCA. Nearly 75% of all cases in young athletes occur in basketball, football, and soccer players.^11^ While a number of high‐profile athletes in these respective sports have experienced cardiac arrest in the past, recent cases have brought the issue back into the spotlight in the media. Just this year, Damar Hamlin suffered a cardiac arrest shortly after tackling a wide receiver during an American football game, resulting in the immediate activation of the National Football League's Emergency Action Plan (EAP).^12^ Christian Eriksen, a Danish soccer player, experienced a cardiac arrest in the middle of a Euro 2020 match.^13^ Team staff and physicians immediately administered cardiopulmonary resuscitation (CPR) and used an automated external defibrillator (AED) to restore his heart rhythm. Collegiate basketball player Vince Iwuchukwu suffered a cardiac arrest during a preseason workout and was also successfully resuscitated.^14^

These incidents highlight the importance of having an effective EAP in place and the need for adequate education in CPR and AED use for the team personnel who are most likely to be present during these events, including other athletes, coaches, and training staff. The objectives of this review are to critically examine studies on the rate and incidence of SCA in athletes, discuss which populations may be at highest risk for these conditions, summarize the data evaluating the efficacy of screening practices which aim to prevent SCA in athletes, and propose a call to action that involves increased awareness and training in EAPs, CPR, and AED use for athletes, coaches, and training staff.

## 2 INCIDENCE OF SCA IN ATHLETES

Estimates of the incidence of SCA in athletes vary widely depending on the methodology used. A recent review of 19 studies by Carrington et al. found that the incidence of SCA in athletes ranged from 0.1 to 2 per 100 000 athlete‐years.^1^, ^15^, ^16^, ^17^ However, as assessed by Harmon et al., studies that primarily utilized media reports or catastrophic insurance claims for reporting were found to underestimate the incidence of SCA in athletes. When studies used stringent methodology, including mandatory reporting systems or national databases, the incidence of SCA was found to be ~1:50 000 in college‐aged athletes and ~1:80 000 in high school‐aged athletes.^17^

In comparison, the incidence of SCA in the general population (ages 1−35 years) is reported as between 0.8 and 1.9 per 100 000 person‐years.^18^, ^19^, ^20^ Furthermore, multiple studies have determined that the relative risk of SCA in competitive athletes is significantly higher than non‐athletes, ranging from 2.5 to 3.6‐fold increased relative risk.^21^, ^22^, ^23^ The increased risk of SCA in competitive athletes is largely believed to be related to vigorous physical demand in those with underlying cardiac disease.

A study of over 10 000 predominantly male adolescent athletes from the English Football Association who underwent a cardiac screening program consisting of a health questionnaire, physical examination, electrocardiography (ECG), and echocardiography found the incidence of SCA to be 1 per 14 794 person‐years.^24^ These results suggest that in specific athlete populations, despite extensive preparticipation screening, the risk of SCA remains comparable to those who do not receive such evaluations.

The discrepancy in estimations highlights the need for a national database to report these catastrophic events and obtain accurate data. The Federation International de Football Association (FIFA) has attempted to fill this gap through the creation of the FIFA Sudden Death Registry (FIFA‐SDR). Starting in 2014, the FIFA‐SDR allows for any involved individuals (athletes, coaches, relatives) to report a soccer‐related SCA or SCD event and provide additional information regarding the circumstances, such as the athlete's age, sex, and medical history.^25^ To supplement this information, FIFA also employs a systematic media monitoring process to capture any events that their self‐reporting system may have missed.^25^ Between 2014 and 2018, a total of 617 players from 67 countries suffered from SCA, with only 142 (23%) surviving.^25^ Registries such as the FIFA‐SDR are important tools for understanding the incidence, risk factors, and potential interventions for SCA in athletes. Unfortunately, this only includes cases from “association” football (soccer).

## 3 COMMON CAUSES OF SCA IN ATHLETES

SCA in athletes can be caused by a wide range of cardiac diseases. Historically, hypertrophic cardiomyopathy (HCM) was thought to be the most common underlying condition that led to SCA in athletes.^26^, ^27^, ^28^ A foundational study from 1996, with data revision from 2009, found that nearly 36% of sudden cardiac deaths in athletes were a result of HCM.^28^ However, more recent analyses have determined that a predominant cause of sudden death in athletes is autopsy‐negative sudden unexplained death (AN‐SUD), which is defined as a sudden death with non‐diagnostic autopsy findings.^20^, ^29^, ^30^, ^31^, ^32^, ^33^ Some studies have estimated the incidence of AN‐SUD may be as high as 31%.^34^, ^35^ Other common causes of SCA in athletes include idiopathic left ventricular hypertrophy, arrhythmogenic right ventricular cardiomyopathy (ARVC), congenital coronary artery anomalies, Long QT syndrome (LQTS), Wolff−Parkinson−White syndrome, Marfan syndrome, and myocarditis.

**Figure 1:**
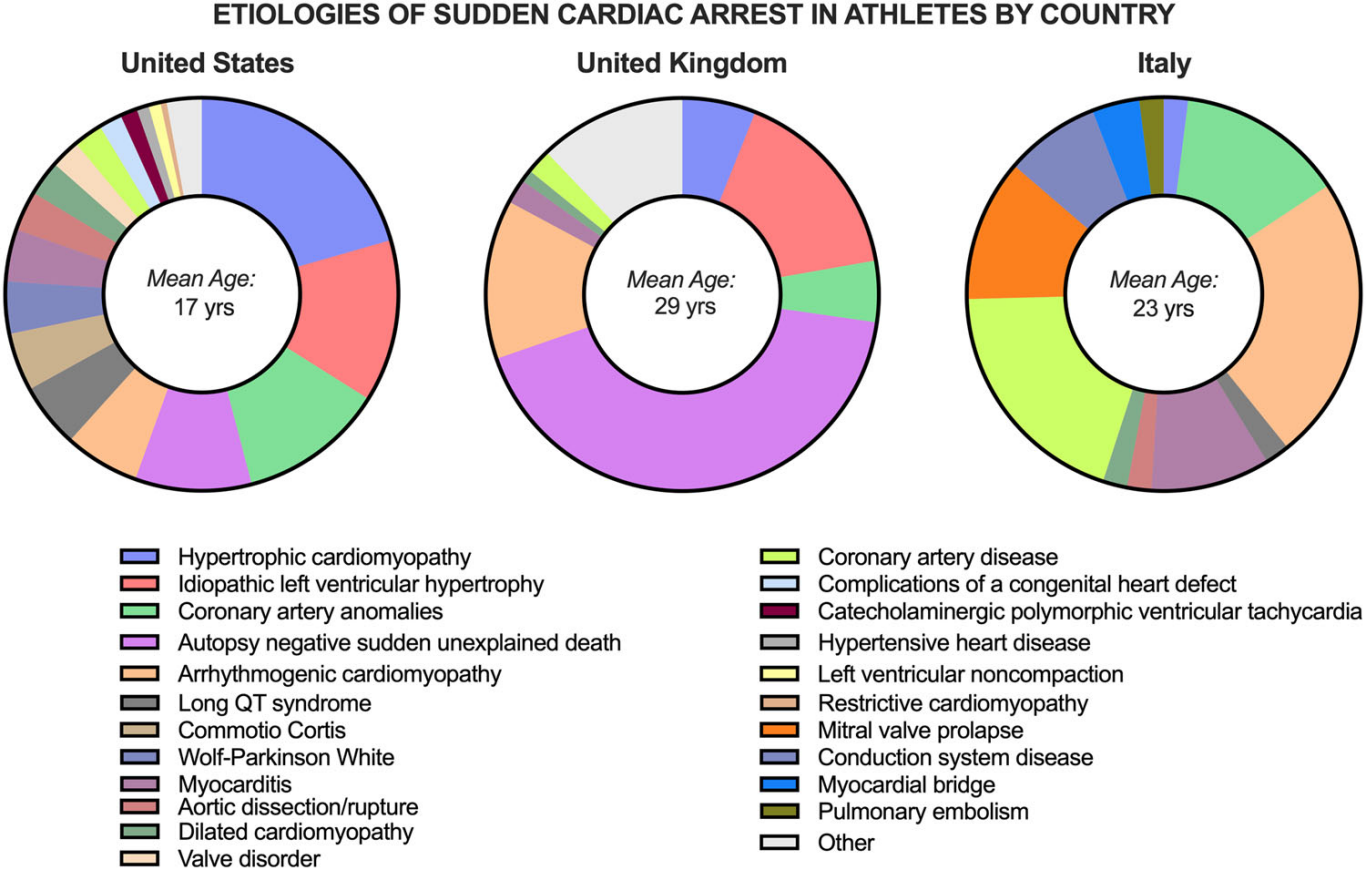
Etiologies of SCA by country in a young athlete population. Comparison of the most common etiologies of SCA in different regions. The data shows that AN‐SUD was the most common cause of SCA in the United Kingdom (mean age 29), while HCM was the most common in the United States (mean age 17). In Italy (mean age 23), ARVC was found to be the most common etiology. This figure highlights the regional differences in the causes of SCA and the importance of understanding the specific etiologies in different populations to improve SCA prevention and management. Figures adapted with permission from Corrado et al.,^22^ Finocchiario et al.,^36^ & and Peterson et al.^37^ ARVC, arrhythmogenic right ventricular cardiomyopathy; HCM, hypertrophic cardiomyopathy; SCA, sudden cardiac arrest.

It is likely that the true incidence of each of these underlying etiologies of SCA varies widely based on certain population characteristics and geographical locations. Figure 1 illustrates the most frequent causes of SCA, categorized by geographical region. In the Veneto region of Italy, ARVC was found to be the most predominant cause of SCA in athletes making up approximately 20% of all cases.^21^ Meanwhile, AN‐SUD accounted for 42% of all sudden deaths in athletes in the United Kingdom.^36^ In the United States, a recent prospective study through the National Center for Catastrophic Sports Injury Research analyzed 331 confirmed SCA/SCD cases and found that the most common causes included HCM (20.6%), idiopathic left ventricular hypertrophy (13.4%), coronary artery anomalies (12.0%), and AN‐SUD (9.6%). When subdividing these cases by age, coronary artery anomalies were found to be more common in middle school athletes (28%), while cardiomyopathies (hypertrophic, arrhythmogenic, dilated, non‐compaction, or restrictive) accounted for 47% of cases in college and professional athletes.^37^

It is suspected that a large portion of the AN‐SUD cases are attributable to cardiac ion channelopathies including LQTS, Brugada syndrome, and catecholaminergic polymorphic ventricular tachycardia (VT).^38^ Given the molecular basis of these disorders, they are unlikely to manifest on traditional pathology and require a genetic autopsy for detection. A prospective study of nearly 500 sudden cardiac death cases in children and young adults found that 27% of AN‐SUD cases had a clinically relevant cardiac gene mutation when genetic autopsy was performed. Moreover, an inherited cardiac disorder was identified in 13% of the families in which AN‐SUD occurred.^18^ These results highlight the need for the consideration of the addition of genetic testing to autopsy investigation to gain a full understanding of the etiologies resulting in SCA.

Notably, these are only some of the common causes of SCA in athletes, and there are many other underlying conditions, such as commotio cordis or aortic dissection, that can lead to SCA. These conditions are most often asymptomatic or may have similar symptoms or presentations, which can make diagnosis difficult. By determining the characteristic etiologies of SCA in certain demographic groups, the optimal method of identifying these underlying conditions can be better defined.

## 4 AT‐RISK POPULATIONS

**Figure 2:**
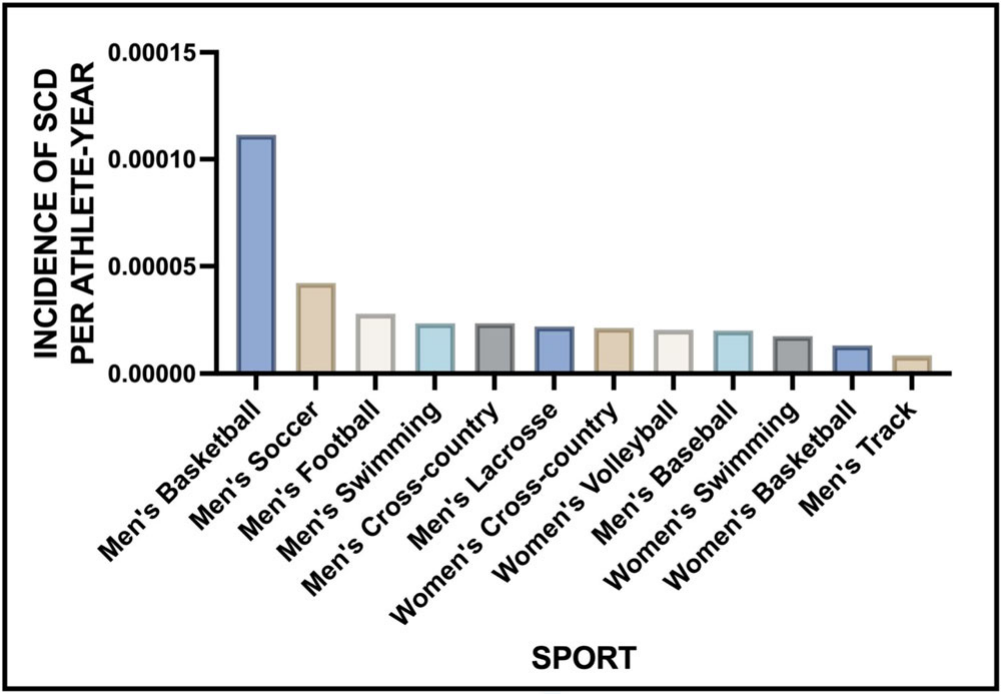
Incidence rates of SCA in athlete‐years by sport. This figure shows the incidence of SCA in different sports based on athlete years. The data reveals that men's basketball had the highest incidence rate, with SCA occurring in 1 out of 8978 athlete years. Men's soccer followed as the next most common sport, with an incidence rate of 1 out of 23 689 athlete years. Figures adapted with permission from Harmon et al.^35^ SCA, sudden cardiac arrest.

Efforts to prevent SCA in athletes have led to attempts to identify the populations most at risk. A decade‐long review of all NCAA deaths performed by Harmon et al.^35^ revealed that male athletes were nearly four times more likely to have an episode of SCA when compared to female athletes (1:37 790 vs. 1:121 593 AY) and Black athletes were nearly three times more likely when compared to their white counterparts (1:21 491 vs. 1:68 354 AY). Additionally, both basketball and football players were found to be at higher risk when compared to athletes from other sports.^1^, ^35^ Figure 2 displays incidence rates of SCA in athlete‐years by sport.

The comprehensive data suggest that the most at‐risk populations include black male basketball and football players.^39^ It has been postulated that the strain placed on the heart during these sports and the higher potential for a marfanoid habitus in the case of basketball player population may contribute to this increased risk. The findings of this study highlight the potential benefits of targeting preparticipation screening, education and awareness campaigns, and cardiac care to these at‐risk populations to help prevent SCA.

## 5 PREPARTICIPATION SCREENING IN ATHLETES

Preparticipation screening in athletes is an important tool for identifying underlying conditions that may lead to SCA. The optimal method for detecting these conditions remains debated, with various guidelines and recommendations from different organizations. The American Heart Association (AHA) guidelines for preparticipation screening currently recommend a targeted 14‐point questionnaire aimed at identifying individuals at the highest risk.^40^ However, recent studies have pointed to the limited sensitivity and specificity of this questionnaire.^41^

As a result, some groups, including the ESC and the IOC, advocate for the addition of the ECG to the history and physical examination.^41^ Proponents of ECG screening point to the success of a landmark study in Italy, in which the rate of SCD declined by nearly 90% after the addition of the ECG to the preparticipation examination.^42^, ^43^ However, a similar study conducted in Israel found no significant difference in the rate of SCD following the implementation of mandatory ECG screening.^44^

The major criticism of ECG screening for athletes is the high number of false positive tests. While the true positive cases are important to identify, the false positive cases result in individuals undergoing additional testing, which may lead to undue cost, unnecessary sports restriction, and anxiety. In the aforementioned Italian study, 9% of all athletes were referred for additional evaluation despite only 2% being determined to have an underlying condition that could predispose to SCD.^42^ These false positives are due in large part to the physiologic adaptations of an athlete's heart that manifest as otherwise abnormal ECG tracings in the general population. Observational studies have been performed to determine the unique ECG findings specific to athletes,^40^, ^45^, ^46^, ^47^, ^48^, ^49^, ^50^, ^51^ and improved characterization of the athlete ECG has led to the development of standardized ECG interpretation criteria, including the 2010 ESC criteria, Seattle criteria, and the now widely accepted international criteria published in 2017.^45^, ^52^, ^53^

The revised criteria have led to a significant reduction in the number of false positives. More specifically, a retrospective study of nearly 5000 athletes screened using the international criteria yielded a false positive rate of only 3%.^54^ Furthermore, an analysis performed by Williams et al. found that the implementation of ECG screening with the International criteria had substantially higher sensitivity (87.5%) and specificity (97.5%) when compared to AHA guidelines alone (18.8% and 68.0%, respectively).^55^

Despite the increasing sensitivity and specificity of preparticipation screening, it is important to consider other factors such as financial, psychological, and the feasibility of widespread implementation. The intricacies of all these intertwined factors have led to the differing recommendations from various organizations, including the AHA, ESC, and IOC. Furthermore, even with mandatory preparticipation screening, episodes of SCA may still occur, albeit at lower rates. As indicated by Robles et al., sport is often carried out outside of a rigid sports infrastructure, and as a result, SCA can occur outside of organized training and competition.^56^ Therefore, it is essential to have readily accessible AEDs and individuals trained in basic life support (BLS) for “*widespread cardioprotection*.”^56^

## 6 EFFICACY OF CPR & AED USE

While the best screening strategies to optimize primary prevention of SCA remains a contested topic, the effectiveness of CPR and AED use in treating SCA has been well‐established. The underlying arrhythmias that can cause SCA include asystole, pulseless electrical activity, pulseless VT, and ventricular fibrillation (VF). VT and VF are particularly responsive to AED treatment, making prompt recognition of these arrhythmias crucial for good outcomes. A study using data from a national French ambulance service found that 47% of sports‐related SCA was caused by either pulseless VT or VF, indicating that a significant proportion of SCA cases have potentially reversible causes.^57^

Studies have revealed that the rapid application of an AED and, specifically, prompt defibrillation when necessary, are key factors in enhancing survival outcomes. For instance, a study by Valenzuela et al. found that 74% of patients with VF survived when defibrillation was performed within 3 minutes, compared to a 49% survival rate for those who had defibrillation performed after 3 minutes.^58^ The importance of bystander initiation is further highlighted by a review of nearly 15 000 episodes of out‐of‐hospital cardiac arrest (OHCA) by Weisfeldt et al., which revealed that survival rates to hospital discharge were 7% with no form of resuscitation attempt, 9% with CPR alone, and 38% when an AED shock was administered.^59^ These data emphasizes the importance of bystander initiation of CPR and AED use in OHCA, as valuable time can be lost waiting for medical personnel to arrive.

## 7 LIMITATIONS OF CPR/AED USE: BYSTANDER INITIATION

Despite the proven benefits of bystander CPR and AED use, their utilization remains low among the general population. According to the Cardiac Arrest Registry to Enhance Survival, only about 40% of bystanders initiate CPR and 6% utilize an AED.^60^ A meta‐analysis of 141 studies conducted across the globe found that only 11.3% of OHCA patients received bystander CPR, despite this subset having better neurological outcomes.^61^ Furthermore, a review of over 28 000 OHCA cases in England found that in non‐EMS witnessed cases, 55.2% received bystander CPR, but only 2.3% utilized public access defibrillation.^62^ The low AED utilization is of particular concern as its use has been shown to have a greater impact on outcomes.

There are disparities in the recipients of bystander CPR and AED use which have been found to be associated with gender, race and/or ethnicity, and socioeconomic status and wealth.^63^, ^64^, ^65^, ^66^, ^67^ For instance, an analysis of nearly 20 000 OHCA found that men were more likely to receive bystander CPR compared to women.^66^ Another retrospective cross‐sectional study conducted in Memphis, Tennessee found that black individuals were less likely to receive bystander CPR compared to their white counterparts. Similarly, those experiencing economic hardship were also less likely to receive bystander CPR.^68^

Exercise‐induced SCA is more likely to present with shockable rhythms such as VT or VF, making the use of an AED imperative for optimal outcomes. However, AED use remains suboptimal in this population.^69^, ^70^ For example, a prospective cohort study conducted in Germany and France found that an AED was utilized in only 7.5% of 147 cases of sports‐related SCA, despite those who received defibrillation having a survival rate of nearly 91%.^70^ In the FIFA‐SDR data, the SCA survival rate was 85% with the use of an AED compared with 35% without, further highlighting the importance of AED utilization.^25^

Multiple studies have suggested that the under‐recognition of SCA is a major factor in the delayed initiation of CPR and AED use.^71^, ^72^ SCA can present with seizure‐like activity and agonal breathing, making it difficult to recognize. In a study by Viskin et al., a video analysis of SCA events found that over 70% of bystanders failed to recognize SCA, with many attempting to open the airway before starting chest compressions.^71^ To improve recognition of SCA, FIFA has implemented practical courses to medical officers, team physicians, and physiotherapists and has also designed mobile application training and treatment videos.^73^

To reduce the number of exercise‐related SCA cases, efforts must be made to increase bystander CPR and AED use through public education campaigns, increasing access to AEDs, and training programs. Additionally, research should be continued to identify, understand, and address any disparities in recipients of CPR and AED therapies.^74^

## 8 INCREASING BYSTANDER/TEAM PERSONNEL CPR/AED USE

**Figure 3:**
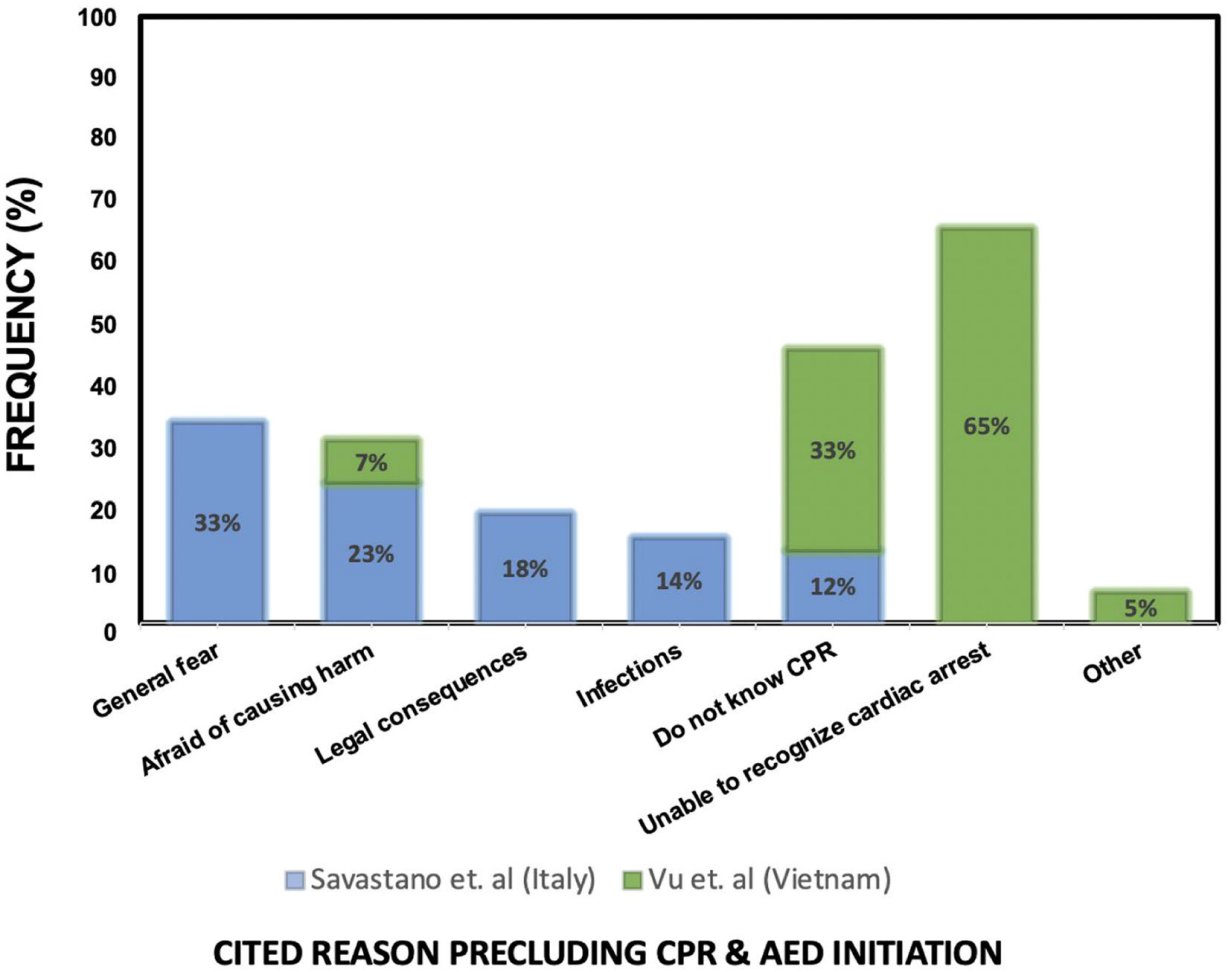
Commonly cited reasons precluding CPR & AED initiation. List of commonly cited reasons that prevent individuals from initiating CPR and using an AED. The most frequently reported reasons include general fear, inability to recognize cardiac arrest, fear of causing harm, and not knowing how to perform CPR. To improve CPR and AED use, addressing these barriers can be aided by increasing public awareness and education about CPR and AED use, as well as providing hands‐on training opportunities to build confidence in performing CPR and using an AED. AED, automated external defibrillator; CPR, cardiopulmonary resuscitation.

To increase the frequency of performance in the general population, it is important to understand the reasons why bystanders often fail to act. Surveys of individuals have found that fear, legal implications, causing harm, and feeling incapable are the most cited reasons for not acting.^75^, ^76^ Figure 3 provides a summary of the most commonly reported reasons why bystanders fail to perform CPR or use an AED. To address these concerns, education and training are key. For example, understanding the Good Samaritan law can alleviate fears of legal repercussions for attempting to revive an individual.^77^ Additionally, providing informational sessions and increasing familiarity with SCA through training team personnel can improve comfort with CPR and AED use.

A study conducted by Ryan et al. discovered that a brief educational and hands‐on training session on using an AED resulted in a significant increase in the number of participants that would utilize an AED in the event of a cardiac emergency.^78^ The study sample consisted of 150 members of sports clubs, aged 16 years and above. The 2 hour in‐person training session was found to have improved their knowledge as well, as evidenced by the results of a follow‐up questionnaire. Similarly, an Australian based study revealed that providing BLS and AED training to sports facilities led to a significant improvement in the participants' knowledge regarding the use of AEDs in clinical scenarios, as assessed 6 months after the training.^79^ The program, sponsored by the government, offered AED and emergency planning training led by certified trainers to all sports venues and community organizations. The facilities and organizations were able to apply for the training program. These studies indicate the efficacy of educational efforts and hands‐on training in improving knowledge and confidence with utilizing an AED in cardiac emergencies, particularly at sporting events, where physical exertion places individuals at higher risk for SCA. By providing AED use training to staff, coaches, and athletes, the number of individuals who can effectively respond to SCA is dramatically increased.

In recent years, smartphones have been employed as a means of enhancing bystander response to OHCA. A study conducted in Denmark employed a smartphone application to alert citizen responders within a one‐mile radius of an OHCA.^80^ The findings showed that in 42% of cases, citizens responded more promptly than EMS, leading to significant improvements in CPR initiation and nearly three times greater use of AEDs.^80^ These results indicate that an app‐based notification system may be an effective strategy in augmenting bystander intervention.

Moreover, not only providing an accessible AED but making sure people know where it is located can also improve the rate of performance. This point was demonstrated in a prospective study where defibrillators were installed less than a 2 minute walk apart in three Chicago airport terminals. Over 80% of those who suffered SCA received defibrillation, with 1 year survival at 56% compared to the <5% with traditional rapid response teams.^81^ These results emphasize the importance of not only educating people on AED use, but also ensuring the devices are readily accessible during emergent situations. By strategically placing defibrillators with clear and noticeable signage at sporting venues, staff, athletes, and spectators can be aware of the AED location, thereby increasing their chances of survival during an episode of SCA.

In sum, there are ample data demonstrating the effectiveness of education, training, technology, and accessibility in increasing AED use and consequently improving outcomes.

## 9 EAP

**Figure 4:**
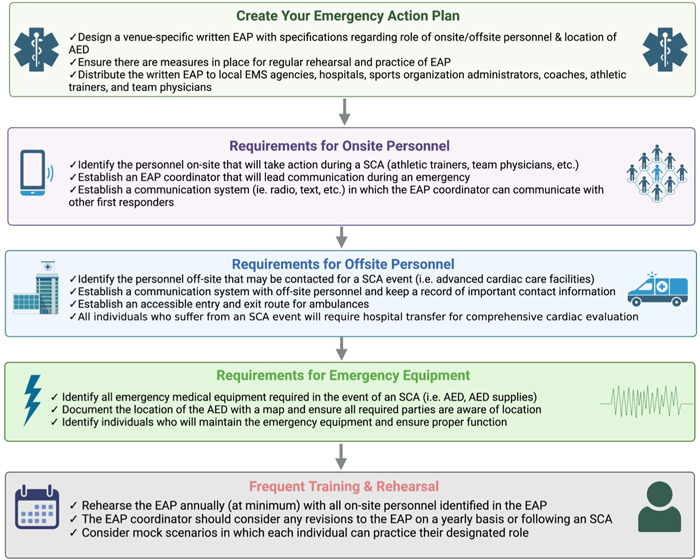
Emergency action plan (EAP) checklist. Checklist for creating a personalized EAP in the event of a sudden cardiac arrest. This includes requirements for on‐site personnel, off‐site personnel, emergency equipment, and rehearsal of the EAP. These are general recommendations following an SCA; site‐specific changes can be made to ensure proper emergency response. SCA, sudden cardiac arrest.

Sports organizations are strongly encouraged by the Interassociation Task Force, AHA, American College of Cardiology, and FIFA to establish an EAP to ensure that SCA events are effectively and properly managed.^82^, ^83^, ^84^ The EAP should include training for personnel on how to respond to a collapsed athlete, easily access an AED, call for emergency medical services, and transport to the nearest advanced care facility.^82^, ^83^, ^84^, ^85^ Figure 4 presents a comprehensive list of the essential components required for an effective EAP.

The essential aspect of the EAP is to train all team personnel and medical staff on how to recognize SCA and respond promptly. SCA should be suspected in all cases where an athlete is unresponsive and in those exhibiting agonal breathing or seizure‐like activity. Once SCA is identified, BLS should be initiated, which includes administering CPR and using the AED. Regular rehearsals of the EAP and precompetition reviews help ensure that individuals are prepared in the event of a cardiac arrest on the field.

In addition to personnel training, the EAP must also take into consideration the specific competition venue. The accessibility of the athlete to bystanders and medical staff may vary, and as such, a different plan of action may be required. Nearby advanced cardiac care facilities may need to be identified, and it is crucial to ensure that an AED is within 3 minutes of the collapsed athlete to improve survival and functional outcomes.^72^

Efficient communication between all participants is also critical to the success of the EAP. The plan should be reviewed and revised regularly, considering feedback from all stakeholders, including medical personnel, athletes, and coaches. In this way, the EAP will remain relevant and effective in ensuring a rapid response to SCA.

Multiple studies have indicated that the implementation of an EAP have directly correlated with an improvement in SCA knowledge and emergency response for young athletes.^86^, ^87^ For example, a recent study was conducted in a youth soccer league where an EAP was implemented through a nurse‐led intervention. Anonymous pre‐ and postintervention surveys indicated that participant knowledge, confidence, and willingness to respond to SCA significantly improved following the intervention.^86^ A similar study found that following two 30 minute training videos regarding EAP implementation and CPR/AED initiation, youth soccer participants had dramatically improved knowledge, perceptions, and decreased fear.^87^

The effectiveness of an EAP has also been demonstrated on the professional level on multiple occasions. The most well‐known example came during the 1996 Summer Olympics in Atlanta, Georgia, when a visiting country's sports delegate suffered from an episode of SCA soon after the games began. Within minutes, athletic trainers and medical staff began CPR on the field and a secondary response team arrived with an AED soon thereafter. The individual was then promptly transported to the stadium medical area where he was successfully intubated and administered cardiac medications before being transported to a nearby hospital.^88^ The rapid and thorough response to the cardiac emergency is a testament to the Olympic organization medical staff's preparedness through the utilization of an EAP. Similar effective responses attributable to the EAP can be seen in the cases of Damar Hamlin and Christian Eriksen. These high profile cases highlight the importance of continuous training on CPR to reduce SCA response time.^89^, ^90^

## 10 CALL TO ACTION: INCREASE TRAINING IN CPR/AED USE FOR TEAM PERSONNEL/COACHES/ATHLETES

**Figure 5:**
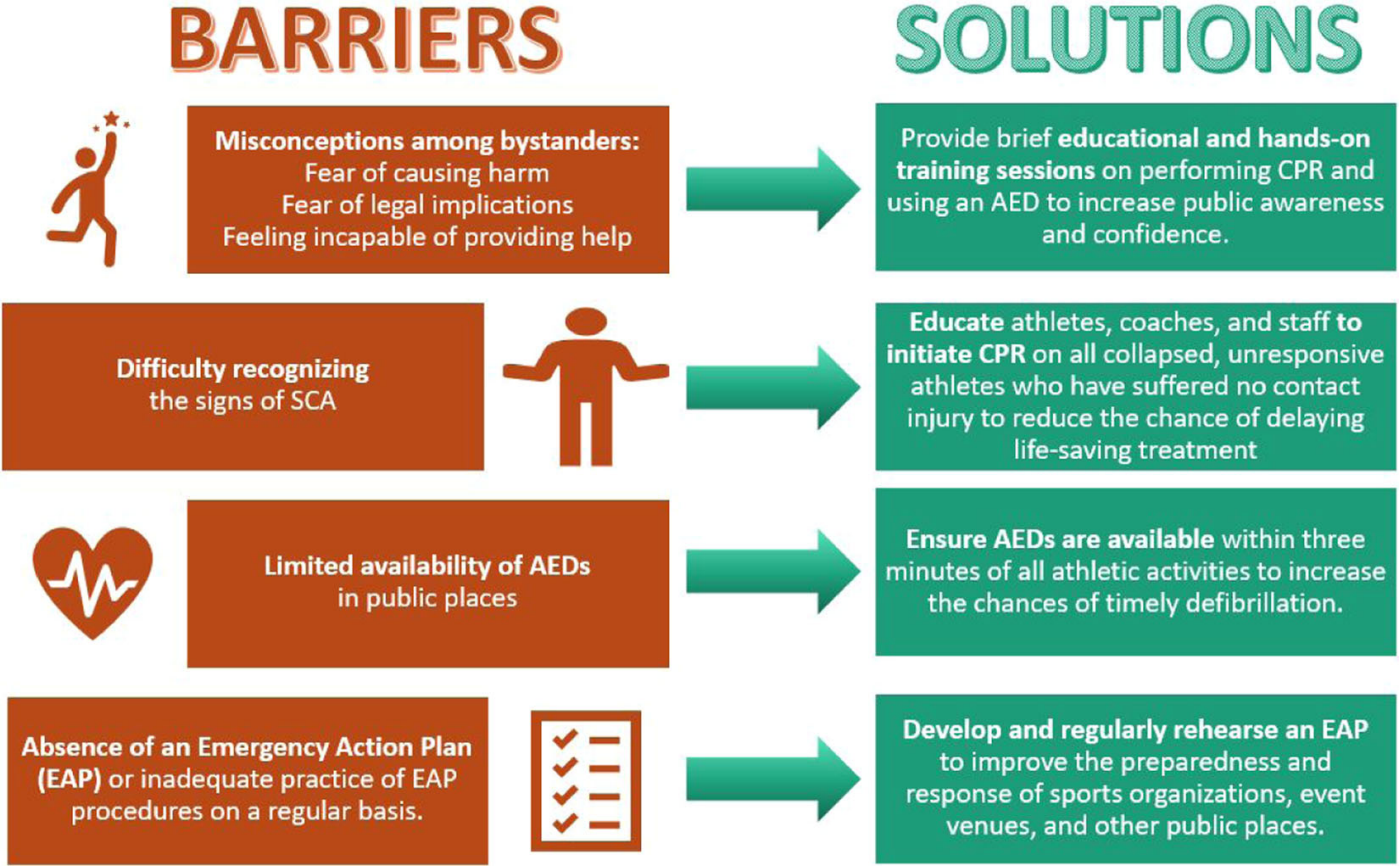
Barriers to optimal care for SCA and possible solutions. The figure illustrates the most common obstacles to providing timely and effective care to SCA patients, which include bystander misconceptions, difficulty in recognizing SCA, lack of availability of AEDs, and the absence of EAP. Possible solutions to address these barriers include educational and hands‐on training sessions, teaching people to recognize signs of SCA, increasing the availability of AEDs around athletic events, and ensuring the presence and rehearsal of EAPs. AED, automated external defibrillator; EAP, emergency action plan; SCA, sudden cardiac arrest.

A crucial element of an EAP for SCA involves communication between three rescuers: the first begins CPR, the second retrieves the nearest AED, and the third contacts emergency medical services. Administering CPR and providing prompt defibrillation, if necessary, are crucial in the survival of the athlete. To ensure that these two steps are completed promptly and correctly, we propose a call to action: increase the teaching of CPR/AED use for athletic training staff, coaches, and athletes. These individuals have the capacity to respond quickly, with athletes demonstrating the quickest reaction times after a FIFA training program.^91^ Figure 5 summarizes the most common barriers to optimal treatment of SCA with potential solutions. Additionally, Table 1 presents a compilation of recommendations for SCA responder and team personnel training, which have been derived from key studies.

**Table 1:** Recommendations for SCA responder training based on key studies.

Reference	Results	Implications
Educational modalities		
Ruppert et al.^92^	Among the 261 medical personnel assessed, the ability to accurately identify breathlessness was found to be only 55.6%.	CPR training should focus more on the determination of breathlessness.
Tsao et al.^60^	Based on data from the Cardiac Arrest Registry to enhance survival, the utilization rates for bystander CPR and AED use were found to be approximately 40% and 6%, respectively.	Highlights the need for increased efforts to educate and empower bystanders to initiate CPR and effectively utilize AEDs, as there is significant room for improvement in bystander response during cardiac arrest events.
Bogue et al.^86^	The implementation of a nurse‐led intervention, which included CPR/AED training and the establishment of an EAP, resulted in a significant improvement in knowledge, confidence, and willingness among participants to respond to SCA.	Nurses are ideal for helping youth sports leagues implement a sustainable SCA action plan based on best‐practice recommendations for emergency health and safety.
Ryan et al.^78^	After participating in a 2 h educational program on AED use, willingness to utilize an AED increased significantly among 142 participants, rising from 20.4% before the education to 77.5%.	A short educational session can significantly enhance individuals' comfort levels and confidence in using AEDs, underscoring the potential value of brief training interventions in promoting effective AED utilization during emergencies.
Vetter et al.^93^	States with laws enacted that require CPR training in high schools have higher rates of bystander CPR following out of hospital cardiac arrest.	Encouraging laws regarding CPR/AED education may result in improved rates of bystander CPR.
Mason et al.^94^	In a survey of youth basketball in the AAU, only 6% of clubs had a written EAP, and 35% required CPR training for coaches.	Efforts should be focused on educating AAU clubs about the importance of EAPs and CPR training to enhance safety measures.
Public policy		
Caffrey et al.^81^	In a prospective study, installing defibrillators within a 2 min walking distance in three Chicago airport terminals resulted in over 80% of SCA cases receiving defibrillation, leading to a 1‐year survival rate of 56% compared to less than 5% with traditional rapid response teams.	Strategically placing defibrillators within close proximity can significantly improve survival rates for SCA cases compared to relying solely on traditional rapid response teams.
Drezner et al.^95^	In a survey of 1710 high schools with an AED program, it was found that 94% of SCA cases received bystander CPR and 83% received a shock from an AED.	The implementation of AED programs in high schools is likely to increase to enhance the utilization of CPR and AED interventions.
Toresdahl et al. ^88^	Prompt recognition, the presence of trained rescuers to initiate CPR, and access to early defibrillation through on‐site AEDs are critical in improving survival from SCA.	All sporting venues should have an EAP for cardiac emergencies. SCA should be suspected for any collapsed and unresponsive athlete to avoid delays in resuscitation.
Andelius et al.^80^	Utilization of a smartphone application to notify citizen responders near OHCA incidents demonstrated that citizen response was faster than EMS in 42% of cases, resulting in notable enhancements in CPR initiation and nearly tripled AED utilization.	Highlights potential benefits of leveraging smartphone applications to engage citizen responders in OHCA situations to increase CPR and AED utilization.

*Note*: This table presents recommendations for team personnel and responder training for SCA, based on significant studies, which are divided into two sections: educational modalities and public policy. These studies have identified potential interventions that can significantly improve survival rates for SCA. The recommendations encompass strategic placement of AEDs, implementation of AED programs in high schools, utilization of smartphone technology, educational sessions focusing on CPR/AED use, and the implementation of legal mandates for CPR/AED training.

Abbreviations: AAU, amateur athletic union; AED, automated external defibrillator; CPR, cardiopulmonary resuscitation; EAP, emergency action plan; EMS, emergency medical services; OHCA, out‐of‐hospital cardiac arrest; SCA, sudden cardiac arrest.

In a prospective observational study of US high schools participating in the National Registry for AED Use in Sports, it was determined that the incidence of SCA was significantly higher in athletes in comparison to the normal population of students, with athletes' relative risk of SCA being 3.65 times higher.^23^ In an evaluation of all cases of sudden death in NCAA student‐athletes across 4 years, it was determined that cardiovascular related sudden death was the leading cause of death, with 75% of those deaths occurring during physical activity.^96^ With the increased risk of SCA in athletes, combined with the risk of the cardiac event occurring during exercise, it can be extremely beneficial to have a trained coach or other athlete in close proximity to the athlete who is able to intervene with CPR/AED use.

Education on proper assessment and diagnosis of SCA should be the first step in CPR training. This knowledge can help athletes recognize when SCA has occurred as opposed to a musculoskeletal or spinal cord injury. An unresponsive athlete who collapsed without any obvious trauma to the head should be assumed to have SCA until proven otherwise. These collapsed athletes may have seizure‐like activity, including myoclonic jerks, and this should not preclude the initiation of CPR or AED use. Occasional gasping following SCA has been mistaken for regular breathing, leading to withholding CPR.^92^ Educating athletes on what qualifies for initiating CPR could eliminate these delays. Half of the cases involving collapse in high school and college athletes showed ongoing respirations or a pulse during SCA.^97^ Therefore, initiatives like forgoing pulse assessment and presuming SCA in the unresponsive athlete is a step forward in reducing delays in CPR.

In a cross‐sectional survey of youth basketball in the Amateur Athletic Union, there were only 6% of clubs with a written EAP and 35% of clubs requiring CPR training for coaches.^94^ Considering the prevalence of SCA in athletes, this lack of proper emergency preparedness for SCA is detrimental to ensuring the survival of athletes. States with laws requiring CPR/AED training in high school have higher rates of bystander CPR following a SCA.^93^ In fact, the use of CPR was trending even higher in 5 of 10 states after the first year of state law enactment, with the greatest improvements coming from communities of lower socioeconomic status.^93^ Lay responders, including athletes and coaches who are trained in CPR, report a greater willingness to attempt resuscitation if faced with an individual experiencing SCA.^98^, ^99^ However, despite evidence that having AEDs readily available increases the chances of survival, only 34% of states require them in some schools in their jurisdictions.^95^, ^100^ Increasing the teaching of CPR/AED use and expanding AED access may happen at the government, sports organization, or even individual level, but ultimately greater awareness and training in CPR and AED use can help save the lives of athletes.

## 11 CONCLUSION

SCA in athletes remains a rare but real risk in young athletes. The formation of formal registries, such as the FIFA‐SDR, will allow for more accurate estimations of SCA in athletes to determine the appropriate public health interventions. Preparticipation screening can play a vital role in reducing the incidence of SCA events, but SCA in athletes will inevitably still occur. However, CPR and AED use have shown to significantly reduce mortality from SCA when executed in a prompt manner. As a result, ensuring athletes and respective staff at all levels have an effective EAP implemented and consistently rehearsed is imperative to improving outcomes from SCA.

The cases of Damar Hamlin, Christian Eriksen, and Vince Iwuchukwu serve as powerful reminders of how an effective EAP can improve the outcome of these catastrophic cardiac events in athletes. Empowering athletes, coaches, and training staff with the knowledge to recognize and respond to SCA can help increase the likelihood of successful stories like theirs.

## AUTHOR CONTRIBUTIONS

**Aneeq Malik**: Conceptualized the project, wrote the manuscript, prepared figures/tables, performed the literature review and analysis, and assisted in detailed revisions. **Justin Hanson**: Wrote the manuscript, performed the literature review and analysis, prepared figures/tables, and assisted in detailed revisions. **Janet Han**: Performed literature review and analysis and assisted in detailed revisions. **Brett Dolezal**: Performed literature review and analysis and assisted in detailed revisions. **Jason S. Bradfield**: Performed literature review and analysis and assisted in detailed revisions. **Noel G. Boyle**: Performed literature review and analysis and assisted in detailed revisions. **Jeffrey J. Hsu**: Conceptualized the project, performed literature review and analysis, and assisted in detailed revisions.

## CONFLICT OF INTEREST STATEMENT

The authors declare no conflict of interest.

## Data Availability

This review article does not present new data, as its primary objective is to critically evaluate and synthesize existing literature. Consequently, there are no specific data sets associated with this article. The conclusions and interpretations drawn in this review are based on the analysis and synthesis of publicly available information and published studies. The references cited in this article provide the sources of data used in the review process. Researchers interested in exploring the data sources referenced in this review are encouraged to consult the original publications cited for further information.
